# Delayed retroperitoneal arterial hemorrhage after inferior vena cava filter deployment: A case report

**DOI:** 10.1097/MD.0000000000009618

**Published:** 2018-01-19

**Authors:** Yongzheng Wang, Haiyang Chang, Wujie Wang, Wei Wang, Bin Liu, Zheng Li, Yuliang Li

**Affiliations:** Department of Intervention Medicine, The Second Hospital of Shandong University, Jinan, China.

**Keywords:** coil embolization, filter, inferior vena cava, pulmonary embolism, thromboembolism

## Abstract

**Rationale:**

Pulmonary embolization is a life-threatening condition. The deployment of inferior vena cava (IVC) filter is the first choice for preventing embolus from the lower extremity. However, IVC filter complications are rare but not to be neglected. Penetration of the arterial wall may result in catastrophic damages. This case report describes a woman who suffered from retroperitoneal hemorrhage after placement of an IVC filter due to a pulmonary embolization. Her filter was found to have penetrated the right lumbar artery and caused the massive bleeding. She was successfully treated with endovascular coil embolization.

**Patient concerns:**

A 47-year old woman presenting with pulmonary embolization was admitted to our hospital. An IVC filter was deployed. Twenty days after her operation, she suffered from an intolerable lumbago when bending over. Contrast-enhanced computed tomography at the local hospital showed a massive retroperitoneal hematoma adjacent to the IVC filter.

**Diagnoses:**

Contrast-enhanced CT at the local hospital showed a massive retroperitoneal hematoma adjacent to the IVC filter. Thereafter, she was transferred to our hospital. Her hemoglobin and INR were 7.1 g/dl and 3.4, respectively. Her systolic blood pressure decreased to 70 mmHg with heart rate increasing to 110 beats/min. The shock index was greater than 1.5.

**Interventions:**

Angiography of the abdominal aorta showed extravasation of contrast medium from the right third lumbar artery. Embolization of the lumbar arteries was performed with coils.

**Outcomes:**

Several days later, she recovered with hematoma shrinking in size and was discharged from the hospital with stable condition.

**Lessons:**

It highlights that appropriate monitoring of patients with IVC filters is an essential part during the long-term management. Endovascular treatment showed a safe and effective way to treat arterial perforation caused by hooks of inferior vena cava filters.

## 1 Introduction

Venous thromboembolism (VTE), encompassing deep venous thrombosis (DVT) and pulmonary embolism (PE), is a common disease with significant morbidity and mortality. PE, in particular, is a life-threatening condition. Inferior vena cava (IVC) ligation for the treatment of DVT was first reported during the early 1970s. This procedure was used with the purpose of preventing PE. Due to the numerous complications associated with ligation, IVC filter placement has been the preferred treatment for the past several decades. However, a systematic review showed that IVC filters was only modestly efficacious in reducing the recurrence of PE ^[1]^. The currently accepted indications for filter deployment include bleeding complications during antithrombotic treatment, patients with contraindications to anticoagulant treatment, floating embolus in the iliac or femoral vein, and thromboembolism recurrences despite optimal anticoagulation.^[2]^ Placement of an IVC filter seems to be a safer choice than surgical plication or ligation for the prevention of PE and can usually be performed with minimal morbidity and mortality. However, occasional complications include penetration of the IVC and duodenum, migration of the filter, and infection.^[3–5]^ Penetration of the arterial wall, although rare, may lead to catastrophic damage.^[6–10]^ We report a case of retroperitoneal arterial hemorrhage due to penetration of the lumbar artery after IVC filer deployment. Percutaneous angiography was performed followed by embolization of the lumbar artery. No extravasation of the contrast medium was detected. The patient recovered well after the intervention and was discharged without symptoms. Percutaneous intervention was proven to be a good option to resolve the IVC filter-caused hemorrhage. The informed consent was obtained from the patient.

## 2 Case presentation

A 47-year-old woman presenting with PE was admitted to our hospital. She suffered from dyspnea, cough, and left chest congestion. She has a medical history of using oral antidepressant agents. Resuscitation was performed immediately before any examination was arranged. Ultrasound of the lower extremity vessels demonstrated DVT in both lower extremities. Contrast-enhanced computed tomography angiography (CTA) revealed a new PE. An IVC filter (Günther Tulip Vena Cava Filter; Cook Medical Inc, Bloomington, IN) was deployed via the right femoral vein with no distinct difficulties. The predeployment angiography showed no malformations in the IVC. She received anticoagulation with warfarin (2.5 mg; Qilu Pharmacy Co Ltd, Jinan, China). The patient recovered well and was discharged with an international normalized ratio (INR) of 2.1.

**Figure 1:**
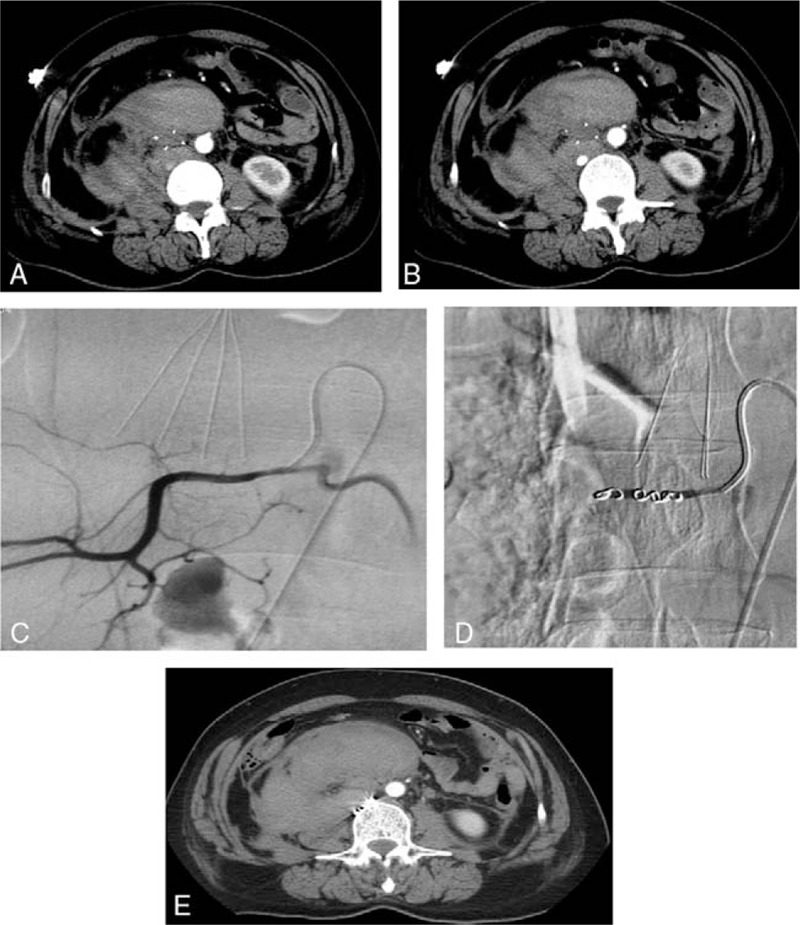
(A and B) The enhanced abdominal CT demonstrated huge retroperitoneal hematoma anterior to the abdominal aorta and adjacent to the filter within the lumen of IVC. (C) The angiography revealed extravasation of contrast medium from the right third lumbar artery due to penetration of the wall caused by the hook of the IVC filter. (D) Coils were deployed in the lumbar artery and post procedural angiogram showed no overflow of the contrast medium. (E) Coils in the artery below the IVC filter were observed on the abdominal CT scan with enhancement. No extravasation of contrast medium was revealed. CT = computed tomography, IVC = inferior vena cava.

Twenty days after her discharge from the hospital, she suffered from an intolerable lumbago when bending over. Contrast-enhanced CT at the local hospital showed a massive retroperitoneal hematoma adjacent to the IVC filter (Fig. 1A and B). Thereafter, she was transferred to our hospital. Her hemoglobin and INR were 7.1 g/dL and 3.4, respectively. Her systolic blood pressure decreased to 70 mm Hg with heart rate increasing to 110 beats/min. The shock index was >1.5. Angiography of the abdominal aorta was performed immediately after resuscitation. The procedure was approved by the ethics committee of the second hospital of Shandong University. Extravasation of contrast medium from the right third lumbar artery was observed (Fig. 1C). Embolization of the third lumbar artery was performed with coils (MWCE-18–3.0–3-HILAL × 2, MWCE-18–2.2–2-HILAL × 1, Hilal Embolization Microcoil; Cook Medical Inc). The second lumbar artery was also occluded with coils (MWCE-18–2.2–2-HILAL × 1, Hilal Embolization Microcoil; Cook Medical Inc) to prevent formation of a collateral circulation (Fig. 1D). Abdominal aorta angiography demonstrated no overflow of the contrast medium. IVC venography was subsequently performed and no abnormalities were observed. She was prescribed bed rest for 2 days. Several days later, she recovered with hematoma shrinking in size (Fig. 1E) and was discharged from the hospital without any symptoms.

## 3 Discussion

VTE is a term that encompasses DVT and PE. In 1884, Rudolph Virchow first proposed that thrombosis was the result of at least 1 of 3 underlying etiological factors: vascular endothelial damage, stasis of blood flow, or hypercoagulability of blood. Risk factors for VTE include increasing age, malignancy, immobility, prior VTE, multiple trauma, and major surgery.^[11]^ It is a common disorder representing a significant source of morbidity and mortality globally. The incidence of VTE is similar to that of myocardial infarction and stroke and the potential benefits of successful prevention are substantial.^[12]^ Although PE is a preventable cause of death, VTE in general is often considered as a complication of another illness, except for a number of specific conditions. Once VTE is diagnosed, the patient should be treated immediately due to that lack of treatment may lead to fatal PE. The most common treatment modality is anticoagulation with low-molecular-weight heparin.

PE is a life-threatening condition. Surgical pressures to prevent acute PE, such as ligation of the femoral vein or IVC, were associated with significant morbidity. Partially occluded clips were later introduced by Adams; however, the incidence of IVC occlusion was 100%.^[13]^ The development of filters in the 1970s provided an effective method of protection from PE with a significantly low rate of IVC occlusion.^[14]^ The filter is employed as a mechanical barrier to prevent embolus originating from the lower or upper extremities from reaching the pulmonary artery and is effective in 98% of patients.^[15]^ Since placement of IVC filters without concurrent administration of antithrombotics poses a high risk of thrombotic occlusion or stenosis, combinatory treatment of anticoagulation, thrombolysis, and filter placement is often used.

Several permanent and retrievable filters are currently available. However, complications arising from their deployment cannot be completely avoided and must be recognized and treated promptly. Immediate complications include mortality (0.16%), infection, heamorrhage, delivery system complications (4%–11%), and venous thrombosis (2%–28%). Remote complications include PE (2%–5%), venous insufficiency (5%–59%), occlusion of the IVC (6%–30%), and filter migration (3%–69%). Rare complications include aortic wall perforation, ureteral injury, duodenum or intestine penetration due to IVC perforation, and symptomatic hydronephrosis.^[15]^ Catastrophic complications are very rare and most complications need no further treatment. Some complications, such as massive hemorrhage, are amenable to percutaneous or endovascular repair.

We report a case of a delayed, symptomatic, and massive retroperitoneal hematoma successfully treated by endovascular repair. Previous reports about retroperitoneal and abdominal cavity hemorrhage after deployment of IVC filters have been published. Almost all of the patients reported were successfully treated with open surgery, except for 1 case that was managed conservatively.^[6–10,15]^ In our case, the acute lumbago, drop in hemoglobin, hypotension, and massive retroperitoneal hematoma around the filter detected by enhanced CT strongly implied arterial hemorrhage caused by the filter. Angiography of the abdominal aorta revealed leakage of the contrast medium from the right lumbar artery, suggesting that the hooks of the filter had penetrated the wall of the IVC and perforated the right lumbar artery. A catheter was introduced into the lumbar artery via an introducer deployed in the right common femoral artery. There was no contrast medium extravasation on repeated angiography after 4 coils had been deployed to occlude the bleeding artery.

## 4 Conclusion

In conclusion, we presented a case of a 47-year-old female with delayed retroperitoneal hematoma caused by an IVC filter penetrating the wall of the lumbar artery. Our own experience as well as our review of the literature suggests that patients with IVC filters, especially those taking anticoagulation drugs, should be monitored closely.
